# Current Status and Future Perspectives of Genomics Research in the Rust Fungi

**DOI:** 10.3390/ijms23179629

**Published:** 2022-08-25

**Authors:** Chongjing Xia, Age Qiu, Meinan Wang, Taiguo Liu, Wanquan Chen, Xianming Chen

**Affiliations:** 1 Wheat Research Institute, School of Life Sciences and Engineering, Southwest University of Science and Technology, Mianyang 621010, China; 2 State Key Laboratory for Biology of Plant Diseases and Insect Pests, Institute of Plant Protection, Chinese Academy of Agricultural Sciences, Beijing 100193, China; 3 Department of Plant Pathology, Washington State University, Pullman, WA 99164-6430, USA; 4 Wheat Health, Genetics, and Quality Research Unit, Agricultural Research Service, U.S. Department of Agriculture, Pullman, WA 99164-6430, USA

**Keywords:** effector, evolution, genomics, rust fungus, secreted protein, whole-genome sequencing

## Abstract

Rust fungi in Pucciniales have caused destructive plant epidemics, have become more aggressive with new virulence, rapidly adapt to new environments, and continually threaten global agriculture. With the rapid advancement of genome sequencing technologies and data analysis tools, genomics research on many of the devastating rust fungi has generated unprecedented insights into various aspects of rust biology. In this review, we first present a summary of the main findings in the genomics of rust fungi related to variations in genome size and gene composition between and within species. Then we show how the genomics of rust fungi has promoted our understanding of the pathogen virulence and population dynamics. Even with great progress, many questions still need to be answered. Therefore, we introduce important perspectives with emphasis on the genome evolution and host adaptation of rust fungi. We believe that the comparative genomics and population genomics of rust fungi will provide a further understanding of the rapid evolution of virulence and will contribute to monitoring the population dynamics for disease management.

## 1. Introduction

Rust is a group of destructive plant diseases. These diseases have caused severe epidemics, damaging crops and even leading to famines in human civilization. Notoriously, many crops of staple foods are subject to rust diseases, e.g., wheat, maize, sorghum, and legumes. Numerous other crops also suffer from rust diseases, including vegetables (e.g., green beans and asparagus), fruits (e.g., apples and pears), ornamentals, (e.g., daylilies and carnations), fiber plants (e.g., cotton and flax), beverage plants (e.g., coffee), and trees (e.g., poplar and pines). Due to the economic importance, extensive efforts have been dedicated to these diseases and many milestones have been achieved in the last century or so [1]. For example, studying wheat, Biffen discovered the resistance to stripe rust following the Mendelian law at the turning point of the last century, beginning with combatting plant diseases through breeding resistant varieties of crops [2]. Working on flax rust, Flor formulated the gene-for-gene hypothesis in the 1940s, which laid the foundation for studying vast pathogen–host interactions [3]. The example of wheat stem rust demonstrated how severe disease epidemics impacted national policies [4]. The emergence and reemergence of more aggressive races, e.g., the Ug99 race group of the stem rust pathogen (*Puccinia graminis tritici*), which was first detected in Uganda in 1998 [5,6], and the coffee leaf rust in Central and South America [7], remind the pathology and breeding programs to continually monitor the rust pathogen populations and breed for resistance. As the Nobel laureate Norman Borlaug said, “Rust never sleeps”.

Rust fungi belong to the order of Pucciniales, class Basidiomycetes in the kingdom of Fungi. These fungi are diverse in life cycles with up to five spore stages, namely pycniospore (or spermatia), aeciospore, urediniospore, teliospore, and basidiospore [8]. If a species has all five spore stages in the life cycle, it is a macrocyclic rust fungus. While a microcyclic rust fungus has teliospore and basidiospore stages (Table 1). Still, other rust fungi, such as *Hemileia vastatrix*, which causes coffee leaf rust, have urediniospore, teliospore, and basidiospore stages but do not produce pycniospores and aeciospores. These rust fungi have hemicyclic life cycles. Most cereal rust fungi are heteroecious, meaning that they rely on two taxonomically different plant hosts to complete their life cycles. For autoecious rust fungi, only one plant host is needed. For the heteroecious rust fungi, the roles of sexual stages on the alternate hosts in disease epidemiology and virulence variation vary greatly in different species depending on environmental conditions as well as alternate host phenology and teliospore dormancy [9]. Since the disease cycle and the five spore stages of the rust fungi have been well reviewed previously [1], we only emphasize several biological features of the rust fungi that are closely related to the current trends of the rust genomics studies.

First, the lack of efficient axenic culture methods makes the rust fungi less tractable and difficult to work with. The rust fungi are obligate biotrophs, depending on the living tissues of host plants for growth and reproduction. Even though very few limited rust fungi can be cultured axenically [20], the mycelia produced from the axenic culture are aberrant [21]. Thus, the technique is not efficient and feasible for most other rust fungi. Secondly, the most common materials for genomics study, urediniospores, have two nuclei within a cell (dikaryotic), with each nucleus as haploid. The heterogeneous nuclei expose the difficulty for genomic studies, as will be discussed later in this review. Thirdly, the isolates (strains) within species vary greatly in the ability to attack different plant species or genera, e.g., in cereal rust fungi. Within a species, different *formae speciales* are defined according to different species or genera of the primary host that the fungus can attack [22]. Genomic studies at the *forma specialis* level have been initiated to study the evolution of rust fungi, as will be discussed below. Moreover, the isolates (strains) within a *forma specialis* may further vary greatly in virulence to infect different cultivars of the primary host crop. Races are generally not distinguishable based on morphological features [23]. In these cases, races are defined by testing isolates on a set of differential cultivars with known/unknown resistance genes. Monitoring the dynamics of races is essential for forecasting potential epidemics and breeding programs.

The first plant pathogenic fungus was sequenced and published in 2005 [24]. Since then, the number of fungi with genome sequences available has increased rapidly [25]. Some species have several to tens of isolates sequenced. One of the reasons contributing to this rapid increase is the extraordinary advancement in genome sequencing technologies [26]. Comparative and functional genomics on plant pathogenic fungi have begun to shed light on various aspects of fungal biology, such as the host range [27], life cycle [28,29,30], mating behavior [31], the interactions with hosts [32,33], adaptation to hosts [34], and the evolution of virulence [35,36]. Particularly, the rust comparative genomics projects were initiated concomitantly in the first decade of the 21st century at the Broad Institute of Massachusetts Institute of Technology Harvard University and the US Department of Energy Joint Genome Institute (JGI) [37]. The rust genomics communities have generated unprecedented insights into various aspects of rust biology. However, a comprehensive and updated summary of these results from rust genomics is scarce. Even fewer reports have guided our understanding of rust biology for future directions. In this review, we first present a summary of the main findings in the genomics of rust fungi related to variations in genome size and gene composition between and within species. Then we show how the genomics of rust fungi has promoted our understanding of the rust pathogen virulence and population dynamics. We finish this review by introducing important perspectives with emphasis on genome evolution and host adaptation of rust fungi.

## 2. Current Status of Rust Genomics

### 2.1. Overview of Rust Genomes

Rust fungi, compared with most other plant pathogenic fungi, have larger genome sizes. The average genome size was estimated as 305.5 Mb based on flow cytometric data [38]. The sizes of the assembled genomes varied from 53 [39] to 1018 Mb [40] (Table 2). This is much larger than the genome size of the smut fungi (around 20 Mb), close taxa of the rust fungi in Basidiomycota [41,42]. The expanded genomes have also been observed in plant pathogenic Ascomycetes and Oomycetes, e.g., 126 Mb in the wheat powdery mildew *Blumeria graminis* f. sp. *tritici* [43], 81.6 Mb in downy mildew *Hyaloperonospora arabidopsidis* [44], and 240 Mb in *Phytophthora infestans* [33]. Interestingly, these fungi and oomycetes all exhibit biotroph stages, suggesting that the genome expansion might be a convergent evolution towards the biotrophic life cycle. Within the rust fungi, there are large variations in genome sizes among families, genera, species, and even isolates. It should be noted that such variations might be caused by the different sequencing technologies and assembly pipelines. So, the true differences, even though important in comparative genomics to study specific biological questions, remain obscure.

There are large proportions of transposable elements and repetitive sequences in the rust fungi genomes (Table 2). In general, a rust fungus has over 30% repetitive sequences in its genome, with the highest percentage over 74%, in the genome of coffee leaf rust fungus *H. vastatrix*. For comparison, the repetitive sequences in the Ascomycetes and other Basidiomycetes are usually less than 20% of the genome. The contribution of repetitive sequences to the expanded genome size has been reported in the oomycetes. For example, given the similar number of coding genes in the oak sudden death pathogen *Phytophthora ramorum* (65 Mb with 28% repetitive sequences) and the late blight pathogen *P. infestans* (240 Mb with 74% repetitive sequences), both are Oomycetes, the expanded genome of the latter is clearly explained by the expanded repetitive sequences. It is speculated that the lack of genome defense systems in the rust fungi against the invasion of transposable elements is responsible for the expanded repetitive sequences. Such defense systems include the repeat-induced point (RIP) mutation [45,46,47], RNA interference [42,48], and DNA methylation [49]. This hypothesis needs to be tested. Moreover, the activities of transposable elements could contribute to genome evolution and the rapid adaptation to different environments, which is particularly beneficial for asexual plant pathogens [50].

Rust genomes encode a relatively large number of proteins, ranging from 14,000 to 32,000 (Table 2). As in the genome size, there are large variations among families, genera, species, and even isolates. Again, these variations are likely generated from the different genome sequencing technologies and gene model prediction methods. Irrespectively, comparative genomics is still able to draw some general conclusions on the gene complement of the rust fungi. For example, rust fungi harbor large numbers of species-specific genes. As high as 41% and 35% of the proteins in the *P. graminis* f. sp. *tritici* and *M. larici-populina* genomes, respectively, did not show sequence similarities in the protein database [28]. Although the species-specific genes decrease with more rust species sequenced, ~6000 genes are still species-specific when closely related species are compared. Each species harbors ~3000 genes that do not share any similarities with the genes in the updated databases [51,52,53]. Most of these species-specific genes do not have functional domains, therefore with unknown functions. Secreted protein-coding genes are of special interest among the species-specific genes, which will be discussed below.

**Table 2:** Genomes of rust pathogens reviewed in this review.

Species ^a^	Strain	Genome Size	No. of Scaffolds	Scaffolds N50/Mb	GC (%)	Coding Genes	Secreted Proteins	Repeat (%)	Reference
*Hv*	HvHybrid	333 ^c^	302,466 ^d^	0.01	33	14,445	483	74.4	[54]
*Hv*	Hv33	549.56	118,162 ^d^	0.009	33.6	13,364	615	43.6	[55]
*Ml*	CH5	189.5	21,310	0.031	41	16,271	1085	45	[56]
*Mlp*	98AG31	101.1	462	1.1	41	16,399	1184	45	[28]
*Pca*	12SD80	99.2	603 ^d^	0.268	44.7	17,248	1532	52.76	[57]
*Pca*	12NC29	105.2	777 ^d^	0.217	44.7	17,865	1548	53.66	[57]
*Pca*	203	101.69	18 ^b^	5.8	44.64	17,877	1985	56.3	[58]
*Pgt*	CDL 75-36-700-3	88.6	392	0.97	43.3	17,773	1106	45	[28]
*Pgt*	21-0	92.5	21,517	-^f^	-	22,391	1924	-	[59]
*Pgt*	PGTAus-pan	94.54	26,417	0.030	43.42	21,874	-	-	[59]
*Pgt*	59KS19	93.30	28,091 ^d^	0.007	-	18,166	-	-	[60]
*Pgt*	99KS76A-1	107.31	28,502 ^d^	0.006	-	18,777	-	-	[60]
*Ph*	Ph560	206.91	838 ^d^	0.405	41.65	25,543	1450	-	[61]
*Pn*	Ard-01	99.93	11,088	0.013	44.9	16,622			[16]
*Pp*	115012-Mr	103–105	-	-	-	>19,000	-	27	[62]
*Ap*	Au_3	1018.08	66	56.243	33.80	18,875	-	91.55	[43]
*Ps*	RO10H11247	99.62	15,722	0.019	43.14	21,087	1244	32.53	[63]
*Pt*	Race 77	102.20	2651	0.1	46.64	27,678	660	37.49	[52]
*Pt*	Race 106	93.99	7448	0.02	46.62	26,384	620	39.99	[52]
*Pt*	Race 1	135.34	14,818	0.5	46.72	14,880	1358	50.9	[51]
*Pt*	77-1	95.12	49,980 ^d^	0.004	46.71	32,824	-	-	[52]
*Pt*	77-2	97.25	64,740 ^d^	0.004	46.71	32,769	-	-	[52]
*Pt*	77-3	97.33	64,499 ^d^	0.004	46.71	32,894	-	-	[52]
*Pt*	77-4	97.22	64,739 ^d^	0.004	46.71	32,745	-	-	[52]
*Pt*	77-5	97.25	64,735 ^d^	0.004	46.71	32,851	-	-	[52]
*Pt*	77-6	97.22	64,853 ^d^	0.004	46.71	32,822	-	-	[52]
*Pt*	77-7	97.26	64,534 ^d^	0.004	46.71	32,757	-	-	[52]
*Pt*	77-8	96.41	65,595 ^d^	0.004	46.71	32,366	-	-	[52]
*Pt*	77-9	96.14	67,081 ^d^	0.004	46.71	32,211	-	-	[52]
*Pt*	77-10	93.66	49,962 ^d^	0.004	46.71	32,180	-	-	[52]
*Pt*	77-11	93.54	50,331 ^d^	0.004	46.71	32,203	-	-	[52]
*Pt*	77-a	97.02	65,649 ^d^	0.004	46.69	32,772	-	-	[52]
*Pt*	77-A1	97.25	64,580 ^d^	0.004	46.71	32,747	-	-	[52]
*Pt*	Pt76	123.91 ^e^	18 ^b^	1.9 ^d^	46.63	-	-	-	[52]
*Psh*	P3TX-2	77.36 ^e^	562	0.218	44.40	15,976	1624	34.20	[64]
*Pst*	93-210	84.62 ^e^	493	0.295	44.39	16,513	1517	36.03	[64]
*Pst*	104E137A-	83.35 ^e^	156	1.3 ^d^	44.40	15,928	1069	37.28	[65]
*Pst*	CYR34	82.67 ^e^	18 ^b^	0.791 ^d^	44.35	17,095	1571	36.38	[66]
*Pst*	93-210	83.95 ^e^	18 ^b^	0.517	44.39	17,946	1678	35.67	[66]
*Pst*	104 E16 A+ 17+ 33+	80.45 ^e^	18 ^b^	4.77		16,272	-	~40	[67]
*Pst*	PST-78	117.31	9715	0.5	44.43	19,542	2147	31.5	[51]
*Pst*	CY32	130	4283	0.125	44.77	25,288	2092	48.91	[68]
*Pst*	PST-130	64.8	29,178 ^d^	-	-	22,815	1088	-	[39]
*Pst*	11-281	84.75 ^e^	427 ^d^	0.385	44.37	16,869	1829	35.81	[69]

^a^ Refer to the footnote of Table 1. ^b^ Chromosomal level. ^c^ A hybrid assembly of right isolates. ^d^ Contig level. ^e^ The primary genome is presented. ^f^ Data not available.

### 2.2. Genomic Variation

As mentioned, each urediniospore of a rust fungus generally contains two genetically distinct nuclei within a single cell. Until now, all genomic analyses have been conducted from genomes generated from dikaryotic urediniospores. Unlike haploid genomes, variations between the two nuclei represent a level of intra-isolate genetic diversity. This phenomenon is referred to as heterokaryosis, but for consistency with the literature, we refer to it as heterozygosity, such as a diploid organism. There are four types of variations in the rust fungal genomes, including single nucleotide polymorphisms (SNPs), insertions and deletions (InDels), gene presence and absence polymorphisms, and segmental duplications. The intra-isolate heterozygosity is slightly different among different rust species. Zheng et al. detected over 80,000 SNPs within the isolate CYR32 of *Puccinia striiformis* f. sp. *tritici* (*Pst*), the wheat stripe rust fungus [68]. *Pst* has a high heterozygosity rate at 5–8 SNPs/kb with large variations depending on the isolates and SNP-calling methods [39,51,70]. In contrast, *P. triticina* (*Pt*), the wheat leaf rust fungus, and *P. graminis* f. sp. *tritici* (*Pgt*), the wheat and barley stem rust fungi, have relatively small heterozygosity rates at 2–3 SNPs/kb. *Melampsora larici-populina* (*Mlp*), the poplar leaf rust, has the smallest heterozygosity rate at 0.8 SNP/kb [51]. Generally, genic regions have higher heterozygosity rates, around 1.5 times than the intergenic regions. Though mutations are important evolutionary resources for heterozygosity, it is possible that heterozygosity raises from the fusion of haploid nuclei through karyogamy. Regardless of the origin, the heterozygosity provides high genetic diversity for the rust fungi, such as *Pst*, for which the sexual stages are not common [9].

Other types of genomic variations have been reported in several rust fungi, but these have not been studied intensively. These genomic variations include InDels, gene presence and absence, and segmental duplication. In a recent study, Xia et al. estimated around two InDels per kb between the two nuclei of urediniospores in two *P. striiformis* (*Ps*) isolates [70]. In contrast, *Pt* has an average of 0.32 InDels per kb estimated from 2 Indian isolates [52]. The gene presence/absence polymorphisms are studied even less. By mapping ion proton reads to the reference genome PST-78, Xia et al. detected a total of 78 genes with the presence/absence of polymorphisms among 14 US *Pst* isolates. Using the correlation analysis with virulence phenotypes, one *Avr* candidate gene was identified from these 78 genes, highlighting the importance of the gene presence/absence in the virulence change in rust fungi [53]. More recently, Miller et al. (2018) and Schwessinger et al. (2018) detected intra-isolate gene presence/absence polymorphisms in *Pst* and *P. coronate* f. sp. *avenae* (*Pca*), the oat crown rust fungus, showing that the allelic counterpart of a gene is absent in one nucleus [65,71]. It will be interesting to test whether these genes are involved in virulence. Moreover, Kiran et al. detected segmental duplications in both *Pst* and *Pt* genomes [52,72]. However, only small segmental duplication regions, with sizes less than 10 kb, were defined. One major obstacle in identifying these complex genomic variations is the high proportion of repetitive sequences in the rust genomes. Therefore, the complete genomes and the utilization of advanced sequencing technologies (e.g., the accurate long-read HiFi platform) are needed to study the contribution of the complex genomic variation in genome evolution.

### 2.3. Effectors in Rust Genomes

Effectors are usually small proteins or RNAs that are deployed by biotrophic pathogens, including rust fungi, to manipulate the plant defenses and cellular processes to promote invasion [73,74,75]. These effectors are delivered from the fungus to different subcellular locations in the host plants, e.g., apoplast and cytoplasm. At the target locations, these effectors interfere with diverse processes, including host cellular metabolic pathways and signaling cascades, RNA silencing, anti-microbial inhibition, and recognition machinery [76]. In the rust fungi, the effectors are secreted from the haustoria, after the germination of urediniospores and penetration of host stomata. A haustorium is a specialized fungal structure formed by the expansion of an infection hypha and invagination into the plant cell plasma membrane [77,78]. A rust fungus encodes a substantial portion of secreted proteins as effectors, around 10% of the proteome, which is in the upper range of the general proportion of 4–14% and reflects its biotrophic lifestyles [56,79]. There are several common features in the identified effectors in the rust fungi. For example, the effectors are small secreted proteins enriched in cystines and haustorially expressed (Table 3). These common features are used in many rust fungal genomics and transcriptomics studies to identify effector candidates [80]. However, it should be noted that these broad criteria do not guarantee the precise predictions of the effectors since there are exceptional cases [81]. Therefore, the bespoke bioinformatic pipelines encompassing multiple criteria are needed to refine effector prediction. Some of the methods are particularly interesting. Saunders et al. presented a hierarchical clustering approach to identify effectors in the rust fungi, in which the proteins are grouped into families using the Markov clustering method, and then the families are ranked according to their likelihood of being effectors using the hierarchical clustering and effector annotation information [82]. This method has been applied to *Pst* and *Melampsora* *lini* [39,56]. The second approach to identifying effectors was developed by Sperschneider et al. based on machine learning and training on the characterized fungal and oomycetes effectors; this approach has been implemented in the programs EffectorP 2.0 and ApoplastP [80,83]. The latter program was designed to predict effectors localized in apoplasts. An additional approach is to integrate genome-wide association studies (GWAS), quantitative trait locus (QTL) mapping, and even population genomics to discover effectors, particularly the Avr effectors. Several studies will be discussed below.

Regardless of the effector candidate mining methods, a rust fungus has a large effector repertoire ranging from a few hundred to thousands, even much larger than the obligate biotrophic powdery mildew fungi [30,43], but compatible with the hemibiotrophic oomycetes *Phythophthora* spp. [33,84]. This raises the question, as noted by Thordal-Christensen et al. [85], as to why the filamentous plant pathogens have so many effectors. We can provide some insights into this question from the genomics and transcriptomics studies of the rust fungi. First, the heteroecious rust fungi utilize distinct effector arsenals during the infection of the two taxonomically unrelated hosts documented in *Mlp* [86]. In this study, Lorrain et al. observed that 20% of all transcripts detected in the rust-infected poplar and larch were specifically expressed on the respective hosts. Among these host-specifically expressed genes, 17% and 25% were secreted protein-coding genes expressed on larch and popular, respectively. These results clearly demonstrated that some of the effectors are utilized specifically during different host infections by the rust fungi. Secondly, different infection stages require different sets of effectors, described as ‘expression waves’ [87]. During the infection of wheat by *Pst*, manipulation of host immune responses by the fungus followed a sequential and temporal manner, and expression waves were observed for several gene clusters [88]. Thirdly, it is possible that the effectors in the rust fungi may be redundant in function. This functional redundancy has been demonstrated in the smut fungus *Ustilago maydis* [89], but not examined in the rust fungi.

A large proportion of effectors in the rust fungi are genus-, species-, and even isolate-specific. Working in the wheat-*Pgt* pathosystem, Rutter et al. revealed the convergent interactions during the infection of wheat by different isolates [60]. Specifically, during the compatible interaction, the same host pathways, e.g., the salicylic acid response pathway, could be targeted by different sets of effectors from different virulent isolates. This study demonstrated that the effectors in different isolates, and probably also in different species, are under functional convergence. In this way, the pathogen makes a trade-off between retaining virulence and diversifying the effectors to evade host defenses. In *Pst*, Xia et al. speculated that the isolate-specific genes might be the remnants of gene loss since a) these genes are redundant in the pathways and b) these genes have significantly lower expression levels [70]. These studies demonstrated that the rust effectors are under diversifying selections exerted from rapid ‘arms-race’ interactions between the rust fungi and their hosts.

**Table 3:** Characterized effectors in rust fungi.

Effector	Species	Expression in Haustoria	Function	Localization	Reference
RTP1p	*U. fabae*; *U. striatus*	Yes	Protease inhibitor	Cytoplasm	[90]
PpEC23	*P. pachyrhizi*	Yes	Suppress HR and basal defense	Unknown	[91]
PEC6	*P. striiformis* f. sp. *tritici*	Yes	Hamper ROS accumulation and Callose deposition	Nucleus, cytoplasm	[92]
PST02549	*P. striiformis* f. sp. *tritici*	Yes	mRNA processing	P-bodies	[93]
MLP124017	*M. larici-populina*	Yes	Unknown	Nucleus, cytosol	[94]
CTP1	*M. larici-populina*	Yes	Unknown	Chloroplast, mitochondria	[93]
MLP124266	*M. larici-populina*	No	Unknown	Nucleus and cytosol	[95]
MLP124499	*M. larici-populina*	Yes	Unknown	Nucleus and cytosol	[95]
AvrMlp7 ^a^	*M. larici-populina*	Unknown	Avirulence	Unknown	[96]
AvrM	*M. lini*	Yes	Avirulence	Plant cell	[97]
AvrL567	*M. lini*	Yes	Avirulence	Plant cell	[98]
AvrP123	*M. lini*	Yes	Avirulence	Plant cell	[97]
AvrP4	*M. lini*	Yes	Avirulence	Plant cell	[97]
AvrL2	*M. lini*	Unknown	Avirulence	Unknown	[99]
AvrM14	*M. lini*	Unknown	Avirulence	Unknown	[99]
PGTAUSPE10-1	*P. graminis* f. sp. *tritici*	Yes	Avirulence	Unknown	[100]
AvrSr27	*P. graminis* f. sp. *tritici*	Yes	Avirulence	Unknown	[101]
AvrSr35	*P. graminis* f. sp. *tritici*	Unknown	Avirulence	Unknown	[102]
AvrSr50	*P. graminis* f. sp. *tritici*	Yes	Avirulence	Unknown	[103]
PSTha5a23	*P. striiformis* f. sp. *tritici*	Yes	PTI suppression	Cytoplasm	[104]
PstSCR1	*P. striiformis* f. sp. *tritici*	Yes	Activate plant immunity	Apoplast	[105]
Pst_12806	*P. striiformis* f. sp. *tritici*	Yes	Suppress HR and basal immunity	Chloroplast	[106]
PstGSRE1	*P. striiformis* f. sp. *tritici*	Unknown	Suppress programmed cell death	Cytoplasm and nucleus	[107]
PstGSRE4	*P. striiformis* f. sp. *tritici*	Yes	Suppress HR	Cytoplasm	[108]
AvrRppK	*P. polysora*	Unknown	Avirulence/PTI suppression	Unknown	[109]
PSTG_01766	*P. striiformis* f. sp. *tritici*	Unknown	Suppress high-temperature seedling resistance	Nucleus, cytoplasm, and membrane	[110]

^a^ Not experimentally validated.

### 2.4. Avr Gene Identification

The concept of *Avr* gene was first proposed by Flor, who worked on the flax–rust interactions [3]. An *Avr* gene encodes a product that can be recognized by the product of a host resistance gene. The recognition subsequently leads to incompatible interactions in which the pathogen shows avirulence and the host shows resistance. The first group of characterized *Avr* genes in the rust fungi were from *M. lini*, the flax rust fungus (Table 3). These first *Avr* genes in *M. lini* were cloned by Dodds et al. using the genetic map cloning along with co-segregated cDNA probes [98]. Two years later, still in *M. lini*, Cantazariti et al. cloned another three *Avr* genes from the haustorially-expressed secreted proteins using the same population as used by Dodds et al. [97]. Recently the *Avr* gene identification in the rust fungi was significantly accelerated by genomics approaches. For example, again in *M. lini*, over ten thousand markers were obtained for a genetic map construction by application of the restriction site-associated DNA sequencing (RADseq) on the sexual population used in Dodds et al. and Catanzariti et al. [99]. By aligning the co-segregated markers in the genetic map to a reference genome, two *Avr* genes were successfully identified. Then the avirulence functions were validated through transient expression assays. The gene *AvrM14* is of particular interest in that it corresponds to two resistance genes, *M1* and *M4*. The single *Avr* corresponding to multiple resistance genes is not uncommon in rust fungi, e.g., *AvrL567* in the study by Dodds et al. [98]. In fact, this phenomenon has also been found in other fungi, e.g., *Leptosphaeria maculans* [111]. Besides the *M. lini*, the genetic mapping of *Avr* genes coupled with genomics approaches has also been initiated, and high-density genetic maps have been constructed in other rust fungi, such as *Pst* [112] and *Mlp* [113]. In *Pgt*, the *AvrSr35* gene was identified through whole-genome sequencing and comparison of chemically mutagenized mutants with natural isolate [102]. Similarly, analyzing the genome environment of another spontaneous mutant detected a large loss-of-heterozygosity region of the *AvrSr50* [103]. Moreover, comparative genomics integrated with association or correlation analyses have also been used to identify candidate *Avr* genes in the rust fungi [53,112,114]. For example, seven *Avr* loci were identified to be associated with virulence towards fifteen resistance genes in *Pca* [57]. More recently, an *Avr* gene corresponding to *Yr26* was also identified using the genome-wide association analysis [115]. In such approaches, instead of sexual populations, the isolates collected from natural populations are used for genotyping and virulence phenotyping. Together these studies show that genomic approaches are promising in studying *Avr* genes in the obligate biotrophic rust fungi. Furthermore, the genotypes used for virulence gene identification could also be valuable resources for further population and genome evolution analyses (see the sections below) at the same time. We expect that more *Avr* candidates will be functionally validated. The identification of *Avr* candidates will further assist the development of molecular markers for diagnosing and monitoring various virulence genes in the rust pathogen populations.

While only a limited number of *Avr* genes have been functionally characterized (Table 3), the attempts to identify *Avr* genes in the rust fungi have revealed several features that are helpful to guide further studies. Firstly, the inheritance of avirulence/virulence phenotypes is complex in rust fungi. Several sexual populations have been constructed for *Pst*, providing opportunities to investigate the segregation patterns of virulence to specific resistance in this fungus [112,116,117,118]. These studies, and other rust fungi [57], suggested that the inheritance of virulence to a single resistance could be controlled by either a single gene or multiple genes. Meanwhile, the *Avr* genes in rust fungi could be either dominant or recessive. The inheritance is even isolate-dependent for some genes [112]. Secondly, the cloned *Avr* genes have shown multiple mechanisms underlying the transition from avirulence to virulence phenotype. The most common one is amino acid changes in Avr proteins caused by non-synonymous substitutions of *Avr* genes, e.g., *AvrSr35* [102]. It is worthy to note that the AvrSr35 protein has an exceptional length of 578 amino acids, which is longer than the length of typical effectors. *AvrSr50* exemplifies another mechanism in which the virulence gene arises through the insertion of a DNA segment (~26.8 Kb) from an avirulence allele [103]. *AvrSr27* is a more interesting example in which the variation in the *Avr* gene expression level is responsible for the avirulence/virulence phenotypes [101]. These examples also highlight the need to screen more types of polymorphisms to pinpoint *Avr* candidates in the association studies. Thirdly, the *Avr* genes of cereal rust fungi reside in the plastic regions in the chromosome. Recent studies have shown that many rust *Avr* genes are tightly linked and form clusters in the genome [10,112]. One example is the *AvYr44-AvYr7-AvYr43-AvYrExp2* cluster in *Pst*. More interestingly, after reanalyzing the data using a chromosome-level reference genome, we clearly mapped this gene cluster to the telomeric region (less than 5 Kb to the telomere repeats) of the short arm in Chr7 [66]. Given that the subtelomeric regions in the plant fungal pathogens are usually unstable and contribute to the virulence diversity [119], this suggests that such a plastic region may promote virulence evolving in *Pst* and other rust fungi.

### 2.5. Pathogenomics

Field pathogenomics is a robust and rapid strategy for surveillance and population analysis of emerging and re-emerging pathogens [120]. In pathogenomics studies, infected host tissues are collected from fields, RNA-seq is applied for genotyping, and then population genetic approaches are used to analyze population structure and diversity. In the pathogenomics study by Hubbard et al. [120], 35 *Pst*-infected wheat and four *Pst*-infected triticale samples were collected from the UK in 2013. Phylogenetic and clustering analyses clearly showed that the *Pst* population from 2013 was diverse, and a dramatic shift was detected by comparing it with the pre-2013 populations. Moreover, the non-synonymous SNPs were selected from polymorphic and differentially expressed effector genes to identify putative effector genes that associate with virulence profiles. After filtering, 42 effector genes were identified. The field pathogenomics approach rapidly detected the dramatic shift of the *Pst* population in the UK in 2013 and suggested that the emerging population might be introduced from more diverse exotic *Pst* lineages and rapidly replaced the previous population [120]. The same approach was applied to analyze the *Pst* population in the UK in the following year [121]. Comparing the isolates of 2014 with that of 2013 suggested a new genetic group present in 2014 but not in 2013, and a genetic group from 2013 was displaced in 2014. These results suggested that the population shift occurred between these two years in the UK. More interestingly, the pathogenomics approach could also detect the seasonally specific genotypes, the genotypes being detected only in certain seasons but not the others. Furthermore, several genetic groups of *Pst* isolates showed high degrees of specificity to wheat varieties. In summary, this RNA-seq-based pathogenomics approach has the power to detect the rapid shift of (re)emerging pathogen populations and, therefore, could be used to timely monitor the changes in the pathogen populations.

## 3. Future Perspectives

Since the initial programs on rust fungi genomics, intensive studies have generated a vast body of knowledge on general features of the rust fungi genomes, the genomic basis for biotrophic lifestyle, and molecular mechanisms for infection and interactions with hosts as mentioned above. However, due to the large size and the repetitive nature of the rust fungi genome, the genomes of numerous rust fungi species are still lacking. Even for the rust fungi that have been extensively studied, many fundamental questions are still inconclusive. These questions include—but are not limited to—the composition of genes and the content of transposable elements. We also have a limited understanding of the impacts of the genome architectures on the rapid changes of pathogenicity, the adaptation to the changing environment, etc. Therefore, we propose the following perspectives for future research on rust fungi genomics (Table 4).

### 3.1. Complete Reference Genome

High-quality complete genomes are required for further genomics studies of the rust fungi. Due to the complexity of fungal genomes, only a limited number of fungal genomes are complete [122,123,124,125,126], especially in the rust fungi. The complete genomes can be achieved through the combination of advanced whole-genome sequencing technologies, and optical and genetic mapping methods. Emphasis should be paid to the importance of manual curation of de novo genome assembly and annotation processes, which may introduce errors [37]. The complete fungal genomes from other fungi have revealed many new genomic features that are missing in the fragmented assemblies, such as telomere and centromere regions. These plastic regions may play pivotal roles in the genome integration, evolution, and environmental adaptations [127]. In fact, filamentous plant pathogens have inclinations toward harboring large, plastic genome compartments that are enriched in effectors and repetitive sequences. Such genomic compartments accelerate the fungal pathogen adaptive evolution [119]. Comparing the complete genomes of different rust species and/or different isolates within a species will enable us to identify such plastic genomes and determine their roles in the rapid evolution of the rust fungi. Moreover, the complete genomes of the rust fungi are needed to understand their genome complexities, e.g., to precisely estimate the inter- and intra-species variations of genome sizes and the transposable elements. Some biological questions, such as mating behavior and organization of mating type genes, could only be answered with the availability of complete genomes [31,51].

The advanced long-read sequencing technologies with increased accuracy (e.g., HiFi from PacBio), coupled with the sophisticated haplotype phasing algorithms, make it possible to generate high-quality complete genomes in the rust fungi [128]. One example of the applications with the chromosome-level genome is the demonstration of the telomere location of *Avr* gene cluster in the *Pst* genome [66]. Moreover, the high-quality genomes, coupled with in-depth transcriptomic analyses, will help to decipher the regulatory mechanisms underlying the complex life cycles of rust [129]. So far, chromosome-level genomes are available for three wheat rust fungi, namely *Pst*, *Pgt*, and *Pt* (Table 2), but the genomes of the rust fungi from wild hosts (representing different *formae speciales*) are lacking. Comparative genomics using these genomes will help to evaluate the contributions from more diverse evolutionary forces that might shape the genetic diversity and adaptation of populations of the cereal rust fungi, e.g., hybridization and introgression, which in general are currently underappreciated [130].

### 3.2. Pan-Genomics

So far, almost all the available reference genomes of the rust fungi were each generated from a single isolate. The single reference genome cannot reflect the genetic diversity of a species. Therefore, the pan-genome concept has been proposed and used to study various organisms [131]. This concept was first adopted to study *Streptococcus agalactiae*, a human bacterial pathogen causing neonatal infection, with the aim to explore the intraspecies gene variability [132]. As summarized in this study, the pan-genome of a species “includes a core genome containing genes present in all strains and a dispensable genome composed of genes absent from one or more strains and genes that are unique to each strain” [132]. Although initiated from a human pathogen, the pan-genome concept is also highly relevant and applicable to plant pathogens, as many plant pathogens have highly flexible genomic compositions, e.g., the core and accessory chromosomes in *Fusarium* spp. [17] and *Zymospetoria tritici* [133]. Due to the relatively large genome size of fungal pathogens compared to bacteria, the pan-genomes of only a few fungi are available [131]. In fact, the PGTAus pan-genome database of *Pgt* is only one pan-genome available in the cereal rust fungi [59]. This pan-genome database was constructed from de novo assembles of unmapped reads after mapping only five *Pgt* isolates to a reference genome, PGT21, resulting in 92 Mb with 13 Mb not presented in the reference genome. Of course, this pan-genome should be enlarged by including genomes of more *Pgt* isolates. Similarly, pan-genomes should be established for many other rust fungi.

With the advancement of sequencing technologies, genomes of multiple isolates for a species can be feasibly obtained. We foresee more pan-genomes will be generated. Here, we briefly discuss several directions on how the pan-genome concept could be applied to rust fungi. One of the advantages of a pan-genome is the composition of the core and dispensable genes/segments. The core regions in a pan-genome present in all strains of a pathogen are speculated to be ideal for targets of fungicides development. The strain-specific dispensable regions, on the other hand, are the major contributor to the intraspecific genome plasticity and encode pathogenicity-related genes. So, these regions are important to identify pathogenicity factors and to reveal the pathogenic evolution. Moreover, the pan-genomes display massive gene presence/absence variations (PAV) that are usually underestimated in the analyses based on a single reference genome. These PAVs provide unprecedented opportunities for identifying pathogenicity genes in the rust fungi. For example, evidence has shown that PAVs might be associated with *Avr* genes in *Pst* [53,115]. Further, the novel genes present in the specific lineages are excellent targets for the development of diagnostic tools for pathogen monitoring, even at the *forma specialis* and race levels in the rust fungi [69,131].

Lastly, we highlight two technical factors that might influence the quality of constructing pan-genomes. First, while the selection of a sufficient number of samples is needed, their phylogenetic relationships and genetic diversity should be far and diverse enough to present the complete heterogeneity of the genomes. Second, each single sample genome should be well-assembled and annotated to model the pan-genome. In-depth reviews of other influencing factors and analytical tools are available [131,134].

### 3.3. Genome Evolution

Our understanding of the genome evolution of the rust fungi is still limited. Besides the plastic genome compartments, there are several processes that may drive genome evolution, but these processes have not been explored in the rust fungi. Recently, we illustrated that gene loss might be an underestimated mechanism in shaping the genome during *P. striiformis* adaption to wheat and barley [70]. More studies should be conducted on the contribution of gene expression, genome rearrangement, transposable elements, etc., to drive genome evolution. Another research area is the importance of reproductive modes during the genome evolution of the rust fungi. Most rust fungi can reproduce sexually. However, asexual reproduction could be the predominant or even the only mode in local regions. It will be necessary to investigate how genome evolution and adaptation occurred in the asexual populations of the rust fungi.

It has been documented that the newly emerged rust fungal populations have adapted to the changing climates and can produce more teliospores, such as the *Pst* [135,136]. Further research could be conducted using genomic approaches to identify genomic regions or genes that are associated with such phenotypes. To this end, whole-genome resequencing of diverse populations within the species is needed. Such research is helpful for understanding the impacts of the changing climate on shaping the genome architecture.

Evidence from controlled conditions has proved the existence of somatic hybridization in the rust fungi [137,138,139]. These studies demonstrated that somatic hybridization could also contribute to genome evolution. However, detection of this process was mainly based on the changes in virulence profiles for known resistance genes or urediniospore colors as phenotypic markers. By doing it this way, somatic hybridization, in many cases, is hard to distinguish from sexual hybridization [140]. In the genomic era, the ever-accessible genotyping methods and the sequenced genomes accelerated the detection and begin to reveal details underlying this process [141]. For example, comparing the genomic segments of different *formae speciales* of *B. graminis*, the cereal powdery mildew pathogen revealed the hybridization between *B. graminis* f. sp. *tritici* and f. sp. *secalis* and gave rise to f. sp. *triticale*. Interestingly, the host of *B. graminis* f. sp. *triticale*, Triticale, is also a hybrid between the hosts of the former *formae speciales*, illustrating hybridization between pathogens specialized in different species is a mechanism of adaptation to new crops introduced by agriculture [34]. Particularly in rust fungi, the near-complete phased haplotypes enable identifying the exchanges of genome segments between isolates that give rise to novel virulence lineages of *Pgt* [142]. We foresee more in-depth details underlying hybridization that could be explored, e.g., identification of recombination breakage sites and their genomic environments, with more high-quality haplotype-phased genomes from diverse hosts available.

### 3.4. Multi-Omics in Rust–Host Interactions

The system view of rust–host interactions was initiated using transcriptomic analyses and demonstrated that the gene expression patterns follow temporarily coordinated waves during the rust–host interactions [88]. However, the detailed regulatory mechanisms underlying gene expressions are still unclear. This might involve transcription, post-translation, epigenetic modifications, etc. To this end, multi-omics is needed. In multi-omics, data from genomics, transcriptomics, proteomics, and metabolomics are combined to quantify gene expression, epigenome, transcript abundance, protein expression, and metabolites at the system level, allowing a comprehensive understanding of the pathogen and host biology and their interactions. While such studies are limited, we provide a recent example in which genomics, transcriptomics, and epigenomics are integrated to reveal the folding and dynamics of three-dimensional (3D) genome organization, which are fundamental for eukaryotes executing genome functions, during the developmental transitions in *Pst* [66]. In this study, high-throughput sequencing coupled with chromosome conformation capture (Hi-C) technology was used to reconstruct the 3D genome organization in the urediniospore and germ tube stages. By comparing with gene expression differences, we found that the regulation of gene activities might be independent of the changes in the genome organization. In addition, the chromatin conformation conservation is independent of the genome sequence synteny conservation in the fungi. While further study is needed to untangle this regulatory mechanism, this study provides an example of understanding the rust–host interactions using integrative analyses of multi-omics data.

### 3.5. Population Genomics

Technically, population genetics has been extended from dozens of loci to the genome level with the accessibility to high-throughput genome sequencing data. Accordingly, traditional questions on the demographic processes in population genetics can be answered more robustly using a very large number of genome-wide markers [143]. Such a population genomics study was conducted using 48 isolates of *Pst* collected from Canada [144]. Whole-genome resequencing was used to generate 1,434,899 markers, and 5543 SNPs were used for population genetic analyses. A subset of the isolates with 23 isolates was genotyped simultaneously using 17 microsatellite markers. Population differentiations revealed from the two types of genotyping were consistent. With high-density genome-wide SNPs, this study was able to further detect the existence of recombination events for each genetic lineage, suggesting the capacity for sexual reproduction or somatic recombination in the Canadian *Pst* lineages. Another example of population genomics was from the coffee leaf rust fungus, *H. vastatrix* [145]. In this study, RADseq was used to generate around 19,000 SNPs from 37 isolates. Phylogenetic analysis and population structure analysis detected potential cryptic species complex with host specialization, which was a striking finding since *H. vastatrix* was believed to be clonal before this study, and no sexual stages were observed. Moreover, this study discovered the introgression events through the sharing alleles between lineages. The detection of the (re)emergence and the origin of the rust fungi can be rapidly achieved by whole-genome resequencing of the field isolates and comparing with the historical isolates and isolates from potential origins [146,147]. Demographic history analyses could be performed to estimate the impact of plant domestication and modern agriculture on the evolution of rust fungi. For example, the intentional breeding and deployment of single/major resistance genes have been demonstrated to continuously contribute to the decline of *Pst* effective population sizes [148]. Further studies will be needed to explore whether the deployment of multiple genes and even resistance genes from wild wheat relatives impacts the *Pst* population demography. In summary, such studies have shown the robustness of pathogen population genomics to reveal the demographic processes of the pathogen populations.

In another aspect, comparative population genomics could also be applied to genome evolution analyses, e.g., to decipher rust speciation and host adaptation. Rust fungi are diverse at both the species and *formae speciales* levels, as mentioned above. Diversification of the rust fungi within a species, the formation of *formae speciales*, is not clear. Several evolutionary processes have been proposed, mostly from other pathosystems, including hybridization [34,149], gene gain/loss events [70], amino acid substitution, and the change of gene expression [150]. These processes could be examined by comparative genomics at the population level. Within a *forma specialis*, the rust fungus rapidly evolves to be more aggressive, e.g., more broadly virulent, and more adapted to various environments. More studies utilizing comparative population genomics are needed to decipher the genomic bases of these processes by identifying genes under selection. Further surveys on these identified genes, potentially the effector genes, could be used to monitor the changing of the rust population and to predict new potential epidemics.

## 4. Conclusions

So far, genomics of the rust fungi is still in its infancy. We expect to have more findings and deep understanding of rust fungal genomics in the near future, especially in the areas of population dynamics, genome evolution, and virulence determinants. Population genomics or pathogenomics-based molecular epidemiology will enable us to detect the sources of origin, identify the migration pathways, and monitor (re)emerging populations more efficiently [151]. With more understanding of the molecular interactions between rust fungi and their hosts, it is also plausible to exploit effector-assisted breeding to quickly identify and deploy resistance genes in crops [152]. We foresee the genomic research will enable us to untangle specific biological questions of the rust fungi, including but not limited to: 1) what are the genomic factors that determine the diverse life cycles; 2) to what extent and how does the sexual reproduction contribute to the diversity of the rust populations; 3) what are the regulatory mechanisms (e.g., transcriptional, post-transcriptional, epigenomic modification, etc.) underlying the interactions between the rust fungus and its hosts; 4) what are (and how do) the evolutionary forces and genomic events drive the evolution of rust fungal populations; and 5) how to utilize effectoromics to effectively enhance breeding new cultivars of the host crops with resistance to the rust diseases. In summary, this fundamental knowledge will ultimately help us design more sustainable management strategies to control rust diseases.

## Figures and Tables

**Table 1:** General biology of rust pathogens with genomes available.

Species ^1^	Disease	Primary Host	Alternate Host	Life Cycle	Reference
*Hv*	Leaf rust	Coffee	Unknown	Hemicyclic	[7]
*Ml*	Flax rust	Flax, linseed	NA	Macrocyclic, Autoecious	[10]
*Mlp*	Leaf rust	Poplar	Larch	Macrocyclic, Heteroecious	[11]
*Pca*	Crown rust	Oat	Buckthorn	Macrocyclic, Heteroecious	[12]
*Pgt*	Stem rust	Wheat, barley	Barberry	Macrocyclic, Heteroecious	[13]
*Pt*	Leaf rust	Wheat	*T. speciosissimum,* *I. fumaroides*	Macrocyclic, Heteroecious	[14]
*Ph*	Leaf rust	Barley	*Ornithogalum*, *Leopoldia*, and *Dipcadi* spp.	Macrocyclic, Heteroecious	[15]
*Pn*	Switchgrass rust	Switchgrass	*Euphorbia* spp.	Macrocyclic, Heteroecious	[16]
*Pst*	Stripe rust	Wheat	Barberry, Oregon grape	Macrocyclic, Heteroecious	[17]
*Psh*	Stripe rust	Barley	Barberry	Macrocyclic, Heteroecious	[18]
*Ap*	Myrtle rust	Myrtaceae	Myrtaceae	Macrocyclic, Autoecious ^2^	[19]

^1^*Hv*, *Hemileia vastatrix*; *Ml*, *Melampsora lini*; *Mlp*, *Melampsora larici-populina*; *Pca*, *Puccinia coronate* f. sp. *avenae*; *Pgt*, *Puccinia graminis* f. sp. *tritici*; *Pp*, *Puccinia psidii* sensu lato; *Pt*, *Puccinia triticina*; *Ph*, *Puccinia hordei*; *Pn*, *Puccinia novopanici*; *Pst*, *Puccinia striiformis* f. sp. *tritici*; *Psh*, *Puccinia striiformis* f. sp. *hordei*; *T*, *Thalictrum*; *I*, *Isopyrum*; *Ap*, *Austropuccinia psidii* (syn. *Puccinia psidii*). ^2^ The life cycle of *Ap* is still unclear.

**Table 4:** Perspectives on genomics research of rust fungi.

Research Field	Features	Approaches
Rust fungal biology	Life-cycle transition Fungicide resistance	Multi-omics, transcriptomics, gene regulatory networks
Rust–host interactions	Pathogenicity Host resistance Regulatory mechanisms	Effectoromics, epigenomics, ATAC-seq, GWAS, genetic mapping, Single-cell RNA-seq, single-nuclear RNA-seq
Genome evolution	Genetic variations Hybridization Sexual reproduction Host adaptation	Whole-genome sequencing, genotyping-by-sequencing, next-generation sequencing, long-reads sequencing, comparative genomics
Epidemiology and disease management	Disease/strain diagnosis Disease monitoring Population diversity	Pan-genomics, population genomics; field pathogenomics

## Data Availability

Not applicable.

## Abbreviations

DNA	deoxyribonucleic acid
RNA	ribonucleic acid
Mb	megabase
RIP	repeat induced point mutation
SNPs	single nuclear polymorphisms
InDels	insertions and deletions
CYR	Chinese yellow rust
*Pst*	*Puccinia striiformis* f. sp. *tritici*
*Pt*	*Puccinia triticina*
*Pgt*	*Puccinia graminis* f. sp. *tritici*
*Pca*	*Puccinia coronate* f. sp. *avenae*
*Mlp*	*Melampsora larici-populina*
GWAS	genome-wide association study
QTL	quantitative trait locus
*Avr*	*Avirulence*
*Sr*	*Stem rust*
*Yr*	*Yellow rust*
PAV	presence/absence variation
Hi-C	high-throughput sequencing coupled with chromosome conformation capture
RADseq	restriction site associated DNA sequencing
